# *In vivo* assessment of the permeability of the blood-brain barrier and blood-retinal barrier to fluorescent indoline derivatives in zebrafish

**DOI:** 10.1186/1471-2202-13-101

**Published:** 2012-08-16

**Authors:** Kohei Watanabe, Yuhei Nishimura, Tsuyoshi Nomoto, Noriko Umemoto, Zi Zhang, Beibei Zhang, Junya Kuroyanagi, Yasuhito Shimada, Taichi Shintou, Mie Okano, Takeshi Miyazaki, Takeshi Imamura, Toshio Tanaka

**Affiliations:** 1 Department of Molecular and Cellular Pharmacology, Pharmacogenomics and Pharmacoinformatics, Mie University Graduate School of Medicine, Tsu, Mie, 514-8507, Japan; 2 Mie University Medical Zebrafish Research Center, Tsu, Mie, 514-8507, Japan; 3 Department of Omics Medicine, Mie University Industrial Technology Innovation Institute, Tsu, Mie, 514-8507, Japan; 4 Department of Bioinformatics, Mie University Life Science Research Center, Tsu, Mie, 514-8507, Japan; 5 Corporate R&D Headquarters, Canon Inc, Tokyo, Ohta-ku, 146-8501, Japan; 6, 2-174 Edobashi, Tsu, Mie, 514-8507, Japan

**Keywords:** Blood-brain barrier, Blood-retinal barrier, Zebrafish, Fluorescent indoline derivatives, Transporters

## Abstract

**Background:**

Successful delivery of compounds to the brain and retina is a challenge in the development of therapeutic drugs and imaging agents. This challenge arises because internalization of compounds into the brain and retina is restricted by the blood–brain barrier (BBB) and blood-retinal barrier (BRB), respectively. Simple and reliable *in vivo* assays are necessary to identify compounds that can easily cross the BBB and BRB.

**Methods:**

We developed six fluorescent indoline derivatives (IDs) and examined their ability to cross the BBB and BRB in zebrafish by *in vivo* fluorescence imaging. These fluorescent IDs were administered to live zebrafish by immersing the zebrafish larvae at 7-8 days post fertilization in medium containing the ID, or by intracardiac injection. We also examined the effect of multidrug resistance proteins (MRPs) on the permeability of the BBB and BRB to the ID using MK571, a selective inhibitor of MRPs.

**Results:**

The permeability of these barriers to fluorescent IDs administered by simple immersion was comparable to when administered by intracardiac injection. Thus, this finding supports the validity of drug administration by simple immersion for the assessment of BBB and BRB permeability to fluorescent IDs. Using this zebrafish model, we demonstrated that the length of the methylene chain in these fluorescent IDs significantly affected their ability to cross the BBB and BRB via MRPs.

**Conclusions:**

We demonstrated that *in vivo* assessment of the permeability of the BBB and BRB to fluorescent IDs could be simply and reliably performed using zebrafish. The structure of fluorescent IDs can be flexibly modified and, thus, the permeability of the BBB and BRB to a large number of IDs can be assessed using this zebrafish-based assay. The large amount of data acquired might be useful for *in silico* analysis to elucidate the precise mechanisms underlying the interactions between chemical structure and the efflux transporters at the BBB and BRB. In turn, understanding these mechanisms may lead to the efficient design of compounds targeting the brain and retina.

## Background

Delivery of compounds to the brain and retina has been an immense challenge in the development of therapeutic drugs and imaging agents [1-8]. Although there are several active internalization mechanisms that can shuttle necessary nutrients into the brain and retina, internalization of molecules is restricted by the blood-brain barrier (BBB) and blood-retinal barrier (BRB), respectively, which maintain homeostasis of these organs [2]. An important constituent of the BBB and BRB is the physical barrier formed by tight junctions between endothelial cells to seal the vascular lumen. The penetration of hydrophilic solutes via the intercellular cleft is severely restricted by the tight junction barrier and only lipophilic compounds with low molecular weight can passively diffuse into the brain and retina by a transcellular route [2]. However, most lipophilic molecules that diffuse into the brain and retina are eliminated by active efflux transporters such as P-glycoprotein (Pgp), multidrug resistance proteins (MRPs), and breast cancer resistance protein (BCRP) [1,9]. Since these efflux transporters have broad substrate spectrums, most endogenous and exogenous lipophilic molecules are unable to reach the brain. Indeed, over 98% of small molecules intended for therapeutic use in the central nervous system never reach the market because of their inherent inability to cross the BBB [3].

Simple and reliable *in vivo* assays are important to identify compounds that can permeate the BBB and BRB. A number of techniques are available for *in vivo* measurement of brain uptake, including methods based on equilibrium studies between the blood and brain, and methods based on kinetic parameters [10]. The equilibrium distribution of a compound between the blood and brain is defined as the ratio of the concentration in the brain and blood (logBB) [10]. This parameter depends upon passive diffusion characteristics, transporters at the BBB, metabolism, and differences between the relative drug binding affinity of plasma proteins and brain tissues. Although logBB measurements provide important information about brain permeability, they usually require several animals per time-point and are therefore costly and labor intensive [10]. Positron emission tomography has been shown to be a noninvasive, quantitative approach for evaluating kinetic parameters of the uptake of compounds by the brain through the capture of multi-dimensional images in real time [10]. However, the preparation and stability of tracers are matters of concern [10]. Therefore, if *in vivo* assays for the assessment of the permeability of the BBB and BRB to a compound can be performed in a high-throughput manner, identification of compounds that can easily cross these barriers will be accelerated. Furthermore, the large amount of data obtained from a high-throughput assay can be used for *in silico* analysis, which has been extensively developed and can greatly contribute to designing and predicting compounds able to cross the BBB and BRB.

Recent developments in combinatorial chemistry have enabled the construction of a diversity-oriented fluorescence chemical library [11]. It has been shown that subtle structural modifications in a compound can alter brain permeability [2]. In this study, we prepared six structurally related fluorescent indoline derivatives (IDs) as a minimum set of diverse fluorescent compounds and evaluated their ability to cross the BBB and BRB in live zebrafish larvae. The BBB and BRB of zebrafish are structurally and functionally similar to those of mammals [12-14]. Furthermore, zebrafish have been used successfully to find fluorescent compounds that permeate the BRB [15]. Thus, we used different transparent zebrafish lines to assess the permeability of the BBB and BRB to these fluorescent IDs *in vivo.* We subsequently focused on the substrate specificity of MRPs to identify the structural factors influencing the permeability of the BBB and BRB.

## Results

### Permeability of the BBB to fluorescent IDs in live zebrafish larvae

The structures and fluorescent properties of IDs used in this study are shown in Table 1. Three IDs (ZMB996, ZMC213 and ZMJ018) of different molecular sizes were prepared from 1-ethylindoline. The compounds shared a common rhodanine ring with an acetic acid group. In addition, we prepared related IDs with a propanoic acid group instead of an acetic acid group (ZMC808, ZMB740 and ZMB034).

**Table 1:** Properties of fluorescent IDs used in this study

	**Name**	**Structure**	**MW**	**cLogP**	**Ex / Em**	**FI**
aceticacid group	ZMB996		348.4	2.38	492 / 576	196
	ZMC213		388.5	3.25	498 / 570	253
	ZMJ018		466.6	4.51	492 / 610	114
propanoicacid group	ZMC808		362.5	2.64	501 / 586	139
	ZMB740		402.5	3.51	513 / 588	217
	ZMB034		480.6	4.77	511 / 627	240

MW, molecular weight; cLogP, calculated octanol/water partition coefficient; Ex, maximum fluorescence excitation; Em, maximum fluorescence emission; FI, fluorescence intensity.

**Figure 1:**
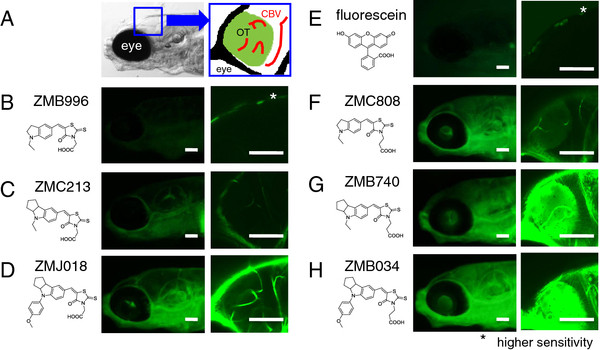
***In vivo *assessment of the permeability of the BBB to fluorescent compounds.** Zebrafish larvae (casper line) at 7–8 dpf were immersed in egg water containing 1 μM of an ID or fluorescein for 1 h. *In vivo* fluorescence imaging of the zebrafish brain was performed using fluorescence microscopes. **A**: Schematic diagram showing the region observed using the fluorescence microscopes. **B-H**: *In vivo* fluorescence imaging of zebrafish larvae stained with ID possessing a rhodanine ring with an acetic acid group (ZMB996, ZMC213, and ZMJ018, **B**, **C** and **D**, respectively), with fluorescein (**E**), and with ID possessing a rhodanine ring with a propanoic acid group (ZMC808, ZMB740, and ZMB034, **F**, **G** and **H**, respectively). The OT was clearly visualized in zebrafish stained with IDs possessing a rhodanine moiety with a propanoic acid group. Scale bar: 100 μm. OT, optic tectum; CBV, cerebral blood vessel.

To examine BBB permeability, we stained zebrafish larvae at 7-8 days post fertilization (dpf) by immersion in medium containing either one of the six IDs or fluorescein (MW: 332; Ex: 494 nm / Em: 521 nm). We focused on the fluorescence signal at the optic tectum (OT), which is a multilaminated structure with a dense neuropil in which tectal cell dendrites receive synapses from many neurons [16] (Figure 1A). Other than in the cerebral blood vessels (CBV), no obvious fluorescence was observed in the OT in zebrafish stained with ZMB996, ZMC213 or ZMJ018, each of which possesses a rhodanine ring with an acetic acid group (Figure 1B-D). While the overall fluorescence signal was extremely low in zebrafish stained with ZMB996 (Figure 1B), weak fluorescence was observed in the CBV of zebrafish stained with ZMC213 (Figure 1C), and strong fluorescence was observed in those stained with ZMJ018 (Figure 1D). The fluorescence signal in the CBV of zebrafish stained with ZMJ018 was much higher than that of ZMC213. In contrast to the staining with the IDs containing a rhodanine ring with an acetic acid group, fluorescence signals were observed in both the OT and CBV in zebrafish stained with ZMC808, ZMB740 and ZMB034, all of which contained a rhodanine ring with a propanoic acid group (Figure 1F-H). The intensity of the fluorescence signal in the CBV and OT from weakest to strongest was obtained with ZMC808 < ZMB740 < ZMB034. This order is consistent with that of the fluorescence intensity (FI) of these dyes (Table 1), suggesting that the intensity of the fluorescence signal *in vivo* reflects the FI of each dye. Another possibility is that ZMB034 and ZMC808 might be the derivatives most and least absorbed by zebrafish, respectively. Neither the OT nor the CBV were stained with fluorescein (Figure 1E).

### Permeability of the BRB to fluorescent IDs in live zebrafish larvae

**Figure 2:**
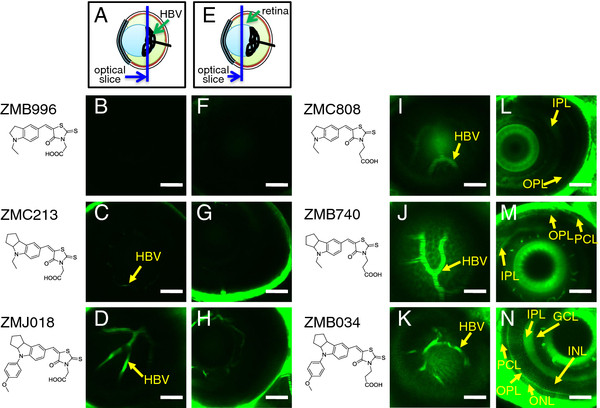
***In vivo *assessment of the permeability of the BRB to fluorescent IDs.** Zebrafish larvae (albino line) at 7–8 dpf were immersed in egg water containing 1 μM of ID. *In vivo* fluorescence imaging of the zebrafish retina was performed using a CLSM. Schematic diagram (**A** and **E**) showing the region of the eye containing the HBV (**B-D, I-K**) and multiple layers of retina (**F-H, L-N**) observed using the CLSM. **B-D** and **F-H**: *In vivo* fluorescence imaging of zebrafish larvae stained with IDs possessing a rhodanine ring with an acetic acid group (ZMB996, ZMC213, and ZMJ018, **B** and **F**, **C** and **G**, **D** and **H**, respectively). The HBV in zebrafish stained with ZMJ018 were clearly visualized. **I-N**: *In vivo* fluorescence imaging of zebrafish larvae stained with IDs possessing a rhodanine ring with an propanoic acid group (ZMC808, ZMB740, and ZMB034, **I** and **L**, **J** and **M**, **K** and **N**, respectively). Both the HBV and multiple layers of retina were clearly visualized in zebrafish stained with ZMB034. Bar: 50 μm. HBV, hyaloid blood vessel; IPL, inner plexiform layer; OPL, outer plexiform layer; PCL, photoreceptor cell layer; GCL, ganglion cell layer; INL, inner nuclear layer; ONL, outer nuclear layer.

Since the BRB and BBB basically share the same barrier system, it is reasonable to assume that compounds that can permeate the BBB can also permeate the BRB. To examine this possibility, we performed *in vivo* imaging of zebrafish retinas stained with the six fluorescent IDs with a rhodanine ring with an acetic acid group or a propanoic acid group (Figure 2). We focused on the fluorescence signal in the hyaloid blood vessels (HBV; Figure 2A-D, I-K) and the multiple layers of the retina (Figure 2E-H, L-N). In the HBV, a strong fluorescence signal was detected in ZMJ018-stained retinas (Figure 2D), a weak signal in ZMC213-stained retinas (Figure 2C), and no signal in ZMB996-stained retinas (Figure 2B). No obvious fluorescence signal was observed in the multiple retinal layers in zebrafish stained with ZMB996 (Figure 2F), ZMC213 (Figure 2G), or ZMJ018 (Figure 2H).

Fluorescence signals were observed in both the HBV and the multiple retinal layers in zebrafish exposed to fluorescent IDs with a rhodanine ring with a propanoic acid group (Figure 2I-N). In zebrafish retina stained with ZMB740 (Figure 2M) and ZMB034 (Figure 2N), fluorescence signals were observed in the inner and outer plexiform layers (IPL and OPL) and the photoreceptor cell layer (PCL), whereas fluorescence in the ganglion cell layer (GCL), inner nuclear layer (INL), and outer nuclear layer (ONL) appeared reticulated. The IPL and OPL are synaptic layers that contain neuronal projections from the INL and GCL, and from the ONL and INL, respectively. The strong fluorescence signals observed in the IPL and OPL (Figure 2N) of zebrafish stained with ZMB034 were consistent with strong fluorescence in the OT where there is an accumulation of synapses (Figure 1H). The fluorescence signal was weak in zebrafish stained with ZMC808 (Figure 2L), which is also consistent with the weak ZMC808-mediated fluorescence signal in the OT (Figure 1F). These results suggest that the permeability of the BRB to fluorescent IDs is similar to that of the BBB.

### Validation of BBB permeability to ZMB034 and ZMBJ018 administered by intracardiac injection

**Figure 3:**
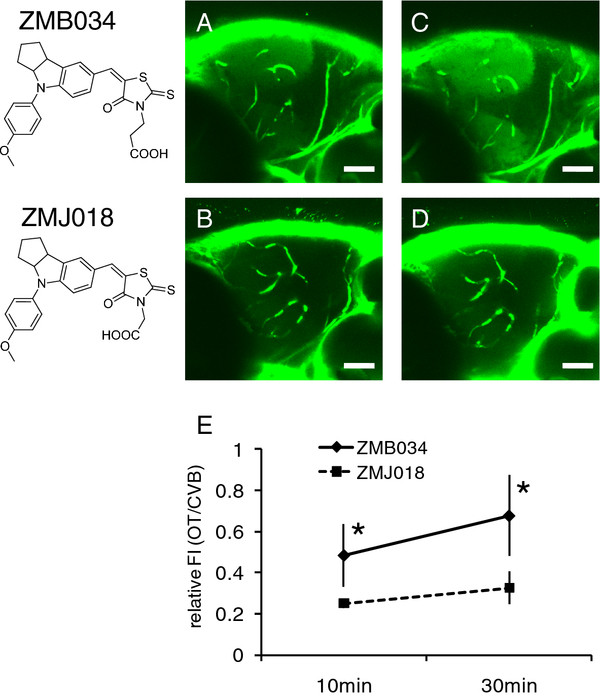
***In vivo *assessment of the permeability of the BBB permeability to ZMB034 and ZMJ018 administered by intracardiac injection.** ZMB034 or ZMJ018 was injected into the cardiac chamber of zebrafish larvae (MK001 line) at 7–8 dpf. *In vivo* imaging of zebrafish brain was performed using a CLSM. **A-D**: *In vivo* fluorescence imaging of zebrafish larvae injected with ZMB034 (**A** and **C**) or ZMJ018 (**B** and **D**) at 10 min (**A** and **B**) and 30 min (**C** and **D**) post-injection. While the CBV were clearly visualized in zebrafish injected with ZMB034 or ZMJ018, the OT was visualized only in zebrafish treated with ZMB034. **E**: Quantitative analysis of FI of the OT relative to the FI of CBV. The relative FI at 10 and 30 min after intracardiac injection was significantly higher in zebrafish injected with ZMB034 than in those injected with ZMJ018 (n = 4, *P < 0.05). Scale bar: 50 μm. FI, fluorescence intensity.

Administration of compounds by immersion might be problematic because the method is likely to be limited to fish and amphibian models. Also, it is not clear whether compounds first enter the blood and then translocate across the BBB and BRB to the brain and retina, respectively. To circumvent these problems, we performed intracardiac injection of ZMB034 or ZMJ018 into zebrafish larvae and assessed the permeability of the BBB to these IDs. As shown in Figure 3, fluorescence signal in the OT was clearly present in zebrafish injected with ZMB034, but not with ZMJ018. The ratio of FI measured in the OT and the CBV of zebrafish injected with ZMB034 increased from 10 to 30 min after injection and was significantly higher than that in zebrafish injected with ZMJ018 at both 10 and 30 min (Figure 3E). These results suggest that the permeability of the BBB to IDs is consistent regardless of either method of exposure.

### Assessment of the structural factors influencing the substrate specificity of MRPs

**Figure 4:**
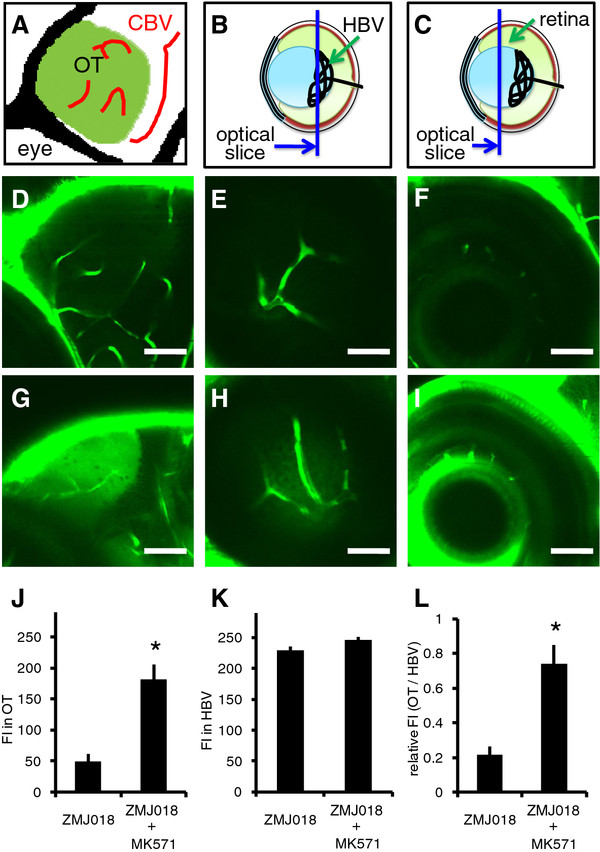
**Effect of MK571 on the permeability of the BBB and BRB to ZMJ018.** Zebrafish larvae (albino line) at 7-8 dpf were immersed in egg water containing 1 μM of ZMJ018 with and without 30 μM of MK571 for 4 h. **A-C**: Schematic diagram showing the regions observed using a CLSM. **D-I**: *In vivo* fluorescence imaging of the OT (**D** and **G**), HBV (**E** and **H**) and multiple layers of the retina (**F** and **I**) in zebrafish stained with ZMJ018 only (**D**-**F**) or ZMJ018 in the presence of MK571 (**G**-**I**). The OT and multiple layers of the retina were clearly visible in zebrafish stained with ZMJ018 in the presence of MK571. **J-L**: Quantitative analysis of the FI in the OT (**J**) and HBV (**K**), and the ratio of the FI (**L**). Both the FI in the OT and the relative FI (OT/HBV) were significantly higher in zebrafish stained with ZMJ018 in the presence of MK571 (n = 4, *P < 0.05). Scale bar: 50 μm. OT, optic tectum; CBV, cerebral blood vessel; HBV, hyaloid blood vessel; FI, fluorescence intensity.

Efflux of compounds from the brain endothelium is initiated at the luminal membrane where ATP-binding cassette (ABC) transporters, including MRPs, BCRP, and Pgp, are the major efflux transporters. MRPs and BCRP largely mediate the efflux of anionic compounds, whereas Pgp mediates mainly the efflux of lipophilic neutral and cationic compounds. According to previous work, MRPs are expressed in various zebrafish organs, including the brain and eyes [17,18]. Because the IDs examined in this study are organic anionic compounds, we examined the effect of the inhibition of MRPs on the permeability of the BBB and BRB to ZMJ018. To inhibit MRPs, we used MK571, a selective inhibitor of MRPs that inhibits the activity of MRPs expressed in zebrafish fibroblast-like cells [18]. Fluorescence signal in the CBV and HBV was observed after staining with ZMJ018 alone (Figure 4D-F). In addition to the CBV and HBV, fluorescence signal was also detected in the OT and multiple layers of the retina in zebrafish stained with ZMJ018 in the presence of MK571 (Figure 4G-I). The FI in the OT of zebrafish treated with both ZMJ018 and MK571 was significantly higher than in those treated with ZMJ018 alone (Figure 4J). The FI in the HBV was similar regardless of MK571 treatment (Figure 4K). The ratio of FI in the OT and the HBV in zebrafish treated with both ZMJ018 and MK571 was also significantly higher than in those treated with ZMJ018 alone (Figure 4L). These results suggest that ZMJ018 is recognized as a substrate and eliminated from the BBB and BRB by MRPs.

## Discussion

### Zebrafish as a useful *in vivo* model for the assessment of BBB and BRB permeability to fluorescent compounds

The zebrafish has been shown to be a useful animal model for studying mechanisms of human disease [19-23], drug discovery for the treatment of human diseases [24], and chemical toxicity [25]. Zebrafish have also been used to research *in vivo* fluorescent imaging agents for the visualization of specific tissues [15]. Accumulating evidence showing similarities between the zebrafish and human BBB and BRB indicates that the zebrafish is a useful research model for studying the development, maintenance, and function of the BBB and BRB in vertebrates [12-14]. Jeong and collaborators [12] injected FITC-dextran (2000 kDa) and rhodamine-dextran (10 kDa) into zebrafish as extraneous markers. Xie and colleagues [13] generated a transgenic zebrafish line that expressed a vitamin D binding protein fused with enhanced green fluorescent protein (DBP-EGFP) in blood plasma as an endogenous tracer. These groups analyzed the leakage of these fluorescent macromolecules from blood vessels and showed that the zebrafish BBB and BRB, which are regulated by tight junction proteins, develop by 3 dpf [12,13].

While similarities exists between the zebrafish and mammalian BBB and BRB [12-14], the contribution of efflux transporters to the function of the zebrafish BBB and BRB is not yet clear. Genes encoding zebrafish homologues of MRPs (*abcc1, abcc4*), MDR (*abcb1*), and BCRP (*abcg2*) have been identified in the zebrafish genome [26]. It has also been shown that *abcc1* mRNA is moderately expressed in the brain and eyes of zebrafish [17]. The present study revealed that treating zebrafish with MK571, a selective inhibitor of MRPs including ABCC1 [27], significantly increased the FI of ZMJ018 staining in both the brain and retina without changing the FI in the CBV and HBV. These results suggest that ZMJ018 is recognized as a substrate and eliminated from the BBB and BRB by MRPs. Thus, efflux transporters in the BBB and BRB may function in zebrafish in a similar manner to those of higher vertebrates. Furthermore, the permeability of a fluorescent ID administered by simple immersion was comparable to that administered by intracardiac injection, supporting the validity of administration of fluorescent compounds to zebrafish by simple immersion for the assessment of BBB and BRB permeability. Used together with a diversity-oriented fluorescence chemical library [11], the combination of these zebrafish-based assays could generate a large amount of information regarding the relationships between the chemical structure and permeability of the BBB and BRB. Such relationships are critical to the development of clinical drugs and imaging agents targeting brain and retina [6,28,29]. To our knowledge, this is the first report demonstrating that zebrafish are an excellent model for the analysis of the permeability of the BBB and BRB to fluorescent compounds, and that this permeability is mediated through efflux transporters.

### Fluorescent IDs as useful compounds for the assessment of BBB and BRB permeability

Fluorescent compounds have been used for assay systems focusing on the interactions of efflux transporters of the BBB in rodent models [29-31]. Fluorescein, a substrate of MRPs, has been used to assess the function of MRPs and the effects of inhibitors of MRPs by measuring fluorescence intensity in rat brain via micro dialysis [30]. It has also been shown that rhodamine 800, a substrate of Pgp, can be used to assess the activity of Pgp in mouse brain by imaging techniques [31]. These findings suggest that fluorescent substrates of efflux transporters are useful for examining the function of transporters in the BBB and BRB in combination with inhibitors of these transporters. If these fluorescent compounds have multiple sites for structural modification that might change their affinity to bind to the transporters, one can reveal important structural features that regulate the permeability of the BBB and BRB. In this study, we took advantage of the indoline scaffold whose structure can be flexibly modified, and prepared six IDs by combining three indoline scaffolds of different sizes and rhodanine derivatives with either acetic acid or propanoic acid. Indoline and rhodanine alone are not fluorescent but the rhodanine ring attached to the indoline scaffold exhibits fluorescence with relatively large Stokes shift [32].

We performed *in vivo* fluorescence imaging of these IDs to analyze the permeability across the BBB and BRB in zebrafish. These IDs were absorbed into the zebrafish from the medium, whereas fluorescein was not. It has been shown that zebrafish can absorb chemicals efficiently from medium into the body when the logP (octanol/water partition coefficient) exceeds 1 [33,34]. This may be the reason why hydrophilic fluorescein was not absorbed by the zebrafish. In contrast, the calculated logP (cLogP) of the IDs ranged from 2 to 5. The fluorescence signal of the IDs in the CBV of living zebrafish corresponded well with their cLogP, suggesting that the quantities of these IDs absorbed in zebrafish may be correlated with their cLogP. Although the lipophilicityof compounds is also an important determinant of their ability to cross the BBB and BRB by passive diffusion [1-8], the lipophilicity of the compounds tested in this study did not correspond with their cLogP. For example, although a strong fluorescence signal was observed in the CBV and HBV of zebrafish stained with ZMJ018, there was no obvious fluorescence signal in the OT and the multiple layers of retina. In contrast, both the OT and the multiple layers of retina were clearly visualized in zebrafish stained with ZMB034 whose cLogP is very similar to that of ZMJ018. These results suggest that the movement of ZMJ018 and ZMB034 across the BBB and BRB may be regulated by mechanisms other than passive diffusion.

Pharmacological intervention using a selective inhibitor of MRPs (MK571) suggested that MRPs pump ZMJ018 across the BBB and BRB from the brain and retina, respectively, to the blood in zebrafish. Comparison of the chemical structure of ZMJ018 and ZMB034 suggests that the number of methylene moieties in the terminal carboxylic acid group attached to the rhodanine ring may be critical for recognition by MRPs. In general, the acidity of carboxylic acid moieties becomes higher as the alkyl substituent becomes shorter. Thus, the acidity of ZMJ018 may be higher than that of ZMB034 because ZMJ018 contains a shorter methylene moiety. Therefore, the recognition of ZMJ018 by MRPs may be greater than that of ZMB034 because MRPs mainly recognize anionic compounds as substrates. Alternatively, the affinity of MRPs for ZMB034 may be less than for ZMJ018 if the dissociation rate of the proton from the terminal carboxylic acid is low. Our preliminary study revealed that fluorescence signals in the CBV, HBV, and OT were similar between zebrafish treated only with ZMB034, and zebrafish treated with ZMB034 and MK571, suggesting that MRPs may have a low affinity for ZMB034. Further studies are required to confirm the interaction between MRPs and these IDs.

In summary, it is possible to synthesize a diversity-oriented library of fluorescent IDs because the substructures can be flexibly modified. These fluorescent IDs can be applied to the zebrafish-based assay because they have a good balance between hydrophobicity and hydrophilicity and can be absorbed into zebrafish from the medium. Thus, the permeability of the BBB and BRB to a large number of fluorescent IDs can be assessed using this zebrafish-based assay.

## Conclusions

We were able to demonstrate that *in vivo* assessment of the permeability of fluorescent IDs across the BBB and BRB could be simply and reliably performed using zebrafish. The substructures of IDs can be modified flexibly and, thus, the ability of a large number of IDs to cross the BBB and BRB can be assessed using this zebrafish-based assay. The large amount of data acquired from the assay might be useful for *in silico* analysis to elucidate the precise mechanisms underlying the interactions between chemical structures and the efflux transporters of the BBB and BRB. A better understanding of the interactions between chemical structures and these efflux transporters will hopefully in turn lead to the efficient design of compounds targeting the brain and retina.

## Methods

### Ethics statement

Mie University Institutional Animal Care and Use Committee guidelines state that no approval is required for experiments using zebrafish. However, animal experiments described in this manuscript conform to the ethical guidelines established by the Institutional Animal Care and Use Committee at Mie University.

### Zebrafish strains

The transparent zebrafish mutant line, *casper*[35], was obtained from the Aquatic Resources Program, Children’s Hospital Boston (Boston, MA) and used for the assessment of BBB permeability (Figure 1). An *albino* zebrafish line [36] was obtained from the Max Planck Institute for Developmental Biology (Tübingen, Germany) and used for the assessment of BRB and BBB permeability (Figures 2 and 4). To study BBB permeability, we also crossed *nacre*[37] and *rose*[38] zebrafish to create MieKomachi 001 (*MK001*), referred to as the *absolute* zebrafish line by the Zebrafish International Resource Center (Figure 3). Zebrafish were bred and maintained according to the methods described by Westerfield [39]. Briefly, zebrafish were raised at 28.5 ± 0.5°C with a 14 h/10 h light/dark cycle. Embryos were obtained via natural mating and cultured in egg water [39].

### Compounds

All fluorescent IDs examined in this study (ZMB034, ZMB740, ZMB996, ZMC213, ZMC808 and ZMJ018) were obtained from Canon Inc. (Tokyo, Japan). Sodium fluorescein and MK571 were purchased from Sigma-Aldrich Co. (St. Louis, MO). Stock solutions (10 mM) of IDs and sodium fluorescein were dissolved in dimethyl sulfoxide (DMSO). Fluorescence excitation and emission spectra and fluorescence intensity of these compounds were obtained by measuring 5 μM solutions of the fluorescent compounds in DMSO with a FL4500 fluorescence spectrophotometer (Hitachi High-Technologies, Tokyo, Japan). MW and cLogP were calculated using ChemDraw 12.0 (CambridgeSoft Corporation, MA).

### Administration of fluorescent compounds into zebrafish larvae

Zebrafish larvae were exposed to each fluorescent ID or sodium fluorescein by immersion in egg water containing 1 μM of the compound for 1 h at 28.5 ± 0.5°C. For the experiments in which MRPs were inhibited, zebrafish were immersed in egg water containing ZMJ018 (1 μM) with or without MK571 (30 μM) for 4 h.

For intracardiac injection of ZMB034 and ZMJ018, zebrafish larvae were anesthetized with 2-phenoxyethanol (500 ppm) and mounted lateral side up in 3% low-melting agarose. Microinjection pipettes were made from glass capillaries (1.0 mm diameter, model GD-1, Narishige, Tokyo, Japan) using a vertical puller (model PC-10, Narishige). The tip of each pipette was broken and then beveled at a 30° angle with a microgrinder (Narishige). Solutions of 3 mM ZMB034 or ZMJ018 in DMSO were loaded into the pipette and approximately 1-2 nl was injected into the ventricle of the beating heart. Thus, the amount of ZMB034 or ZMJ018 delivered into the circulatory system was approximately 3-6 pmol, which is comparable to the amount of fluorescent dextran (10 pmol) injected into the circulatory system for microangiography in zebrafish [40]. Only zebrafish exhibiting fluorescence in CBV at 10 min after injection were analyzed.

There are studies analyzing the amount of compounds in zebrafish immersed in the medium containing the compounds. When zebrafish embryos were immersed in the medium containing 15 μM nicotine for 10 min or 4 μM of PFOS for 1 h, the amount of nicotine or PFOS in the zebrafish embryo was about 10 pmol or 2 pmol, respectively [41,42]. Together, these previous studies suggest that similar concentrations of IDs can be achieved in zebrafish through intracardiac injection of 1-2 nl of a 3 mM solution and immersion in a 1 μM solution for 1 h, which were the treatment strategies used in the present study.

#### *In vivo* assessment of the permeability of the BBB and BRB to fluorescent compounds

After administration of fluorescent compounds, Zebrafish larvae were washed, anesthetized with 2-phenoxyethanol (500 ppm), and transferred onto glass slides. A few drops of 3% methyl-cellulose solution were placed over the larvae and the larvae were immediately oriented on the lateral side. The brain and retina of the embedded larvae were observed using a Leica MZ16 FA fluorescence stereomicroscope (Leica Microsystems, Wezlar, Germany), and a Zeiss 510 confocal laser scanning microscope (CLSM) using a 20× (NA 0.75) objective lens (Carl Zeiss, Oberkochen, Germany) according to a previous report [15]. Excitation was performed at 488 nm and fluorescence emission above 505 nm was detected using an argon laser and a longpass filter, respectively. For the experiments in which IDs were administered by exposure in water, the image acquisition setting was fixed within the same experiment, but the setting varied among different experiments. All images acquired from the CLSM were processed with a Zeiss LSM Image Browser (Carl Zeiss) and Volocity (Perkin Elmer, MA, USA).

### Quantitative analysis of the *in vivo* fluorescence imaging of zebrafish brain and retina

Images were analyzed with ImageJ (http://rsbweb.nih.gov/ij/index.html) to measure FI. Five circular (diameter, 20 μm) regions of interest (ROI) in the OT, which did not include any CBV, were generated in each sample (Figures 3 and 4). Twenty single pixel ROI within CBV (Figure 3) and HBV (Figure 4) were also generated in each sample. The mean intensity of these ROIs (circular or single pixel) was calculated for each sample. Four biological replicates were performed for each experimental condition.

Statistical analysis was performed using SAS version 9.1 (SAS Institute, NC). The Student’s *t*-test was performed to compare the means of two groups. *P* < 0.05 was considered significant.

## Abbreviations

BBB: Blood-brain barrier; BRB: Blood-retinal barrier; IDs: Indoline derivatives; DMSO: Dimethyl sulfoxide; MRPs: Multidrug resistance proteins; Pgp: P-glycoprotein; BCRP: Breast cancer resistance protein; CLSM: Confocal laser scanning microscope; OT: Optic tectum; CBV: Cerebral blood vessel; HBV: Hyaloid blood vessel; FI: Fluorescence intensity; dpf: Days post fertilization; MK001: MieKomachi 001; IPL: Inner plexiform layer; OPL: Outer plexiform layer; PCL: Photoreceptor cell layer; GCL: Ganglion cell layer; INL: Inner nuclear layer; ONL: Outer nuclear layer.

## Competing interests

KW, TN, TS, MO, TM, TI are employed by Canon Inc. TS, TM, KW, TN, TT, YN, and YS developed the indoline derivatives as probes for a biological specimen (patents pending: WO/2010/074326, WO/2011/077751).

## Authors' contributions

KW and YN conceived the study, carried out the experiments and wrote the manuscript. TN, NU, ZZ, BZ, JK, YS, TS and MO helped to carry out the experiments. TM and TI conceived the study. TT conceived the study and wrote the manuscript. All the authors have read and approved the final version of the manuscript.
